# Shikonin and Co^2+^ self-assembled nanoparticles promote diabetic wound healing via antioxidant effects

**DOI:** 10.3389/fchem.2026.1785594

**Published:** 2026-04-22

**Authors:** Xiaoge Wang, Liren Ma, Jinxu Qi

**Affiliations:** 1 Pingdingshan Hospital of Traditional Chinese Medicine, Pingdingshan, China; 2 Medical College, Pingdingshan University, Pingdingshan, China

**Keywords:** cobalt ions, diabetic wounds, epidermal regeneration, nanoparticles, shikonin

## Abstract

This study successfully constructed shikonin-Co nanoparticles (Co-Shik NPs) and systematically evaluated their healing effects on diabetic wounds. The nanoparticles, prepared via a self-assembly method, exhibited uniform particle size and stable structure. *In vitro* experiments demonstrated that the material showed no cytotoxicity at a concentration of 16 mg/L, significantly scavenged reactive oxygen species, and reduced the H_2_O_2_-induced apoptosis rate from 25.1% to 10.31%. Animal experiments revealed that the nanoparticle-treated group achieved a wound healing rate of 95% by day 12, which was significantly superior to the control group. Co-Shik NPs effectively modulated inflammatory factors (reducing IL-1β and TNF-α, elevating IL-10), alleviated oxidative stress, and promoted collagen deposition and epidermal regeneration. This study provides a novel material for diabetic wound treatment, deepens the understanding of the biological activities of natural product-metal complexes, and holds significant theoretical value and application prospects.

## 1 Introduction

Skin wound healing is a complex and continuous process (Guo et al., 2025; Koga et al., 2025; Mohanty et al., 2026). When the local wound microenvironment is disrupted by external factors or intrinsic physiological conditions, healing is often delayed, potentially leading to the formation of chronic wounds (Xiao et al., 2025; Zhang X. et al., 2025; Zhang Y. et al., 2025). In diabetic patients, factors such as a hyperglycaemic environment, excessive oxidative stress, and imbalanced inflammatory responses impede wound repair within the normal timeframe, making the wounds prone to developing into diabetic chronic wounds (Wang et al., 2024; Wang et al., 2025; Yampolsky et al., 2024). In diabetic wounds, hyperglycaemia and impaired vascular homeostasis lead to tissue hypoxia, triggering excessive oxidative stress (Ahmed et al., 2024; Hong et al., 2025). The accumulation of surplus ROS further exacerbates tissue inflammation. Therefore, modulating the redox balance and scavenging excess ROS have become key strategies for promoting the healing of diabetic wounds (Ahmed et al., 2025; Schmidt et al., 2025; Zhang J. et al., 2025).

Natural compounds have garnered significant attention in wound treatment due to their multiple biological activities (Falbo et al., 2023; Gaspar-Pintiliescu et al., 2019; Zhao et al., 2025). Shikonin (Shik), a natural bioactive component with anti-inflammatory, antioxidant, and antibacterial properties, has been shown to reduce ROS generation and alleviate oxidative stress by regulating cellular signalling pathways, such as the NRF2 pathway (Deng et al., 2026; Han et al., 2023; Shu et al., 2022). Simultaneously, it modulates the expression of inflammatory factors and promotes the polarisation of macrophages towards the reparative M2 phenotype (Bai et al., 2024; Luo et al., 2025). Furthermore, Shik can enhance the migration and proliferation of keratinocytes and fibroblasts, thereby accelerating re-epithelialisation and collagen deposition (Ding et al., 2024; Li et al., 2025; Xue et al., 2024). However, the poor water solubility, low bioavailability, and potential toxicity of Shik limit its direct application, creating an urgent need for novel delivery systems to achieve its controlled release, enhanced efficacy, and reduced toxicity. Metal ions (e.g., silver, zinc, copper) have been widely utilised in diabetic wound dressings due to their antibacterial, pro-angiogenic, and antioxidant properties (Hacimuftuoglu et al., 2024; Liu et al., 2024; Nadaroglu et al., 2017a; Nadaroglu et al., 2017b; Shrestha et al., 2024; Sukhodub et al., 2023). For instance, cobalt (Co) ions can activate the HIF-1α signalling pathway by mimicking a hypoxic environment, promoting the expression of vascular endothelial growth factor (VEGF) and improving local ischaemia (Sharda and Choudhury, 2023; Shi et al., 2019; Vasilyeva et al., 2025). Nonetheless, issues such as the biological toxicity and uncontrolled burst release of metal ions constrain their clinical application. Integrating metal ions with natural active ingredients via nanotechnology to construct composite systems with synergistic functionality and controlled release represents a current research focus in diabetic wound therapy (Mani et al., 2025; Nqakala et al., 2021; Singh et al., 2025).

In this study, we rationally designed and prepared Co-Shik self-assembled nanoparticles for type 1 diabetic wound repair. Compared with individual shikonin or cobalt ions, this self-assembled system exhibits improved stability and biocompatibility, and exerts potent therapeutic effects mainly through enhanced antioxidant activity to alleviate oxidative stress and accelerate wound healing.

## 2 Results and discussion

### 2.1 Preparation and characterisation of Co-Shik NPs

**FIGURE 1:**
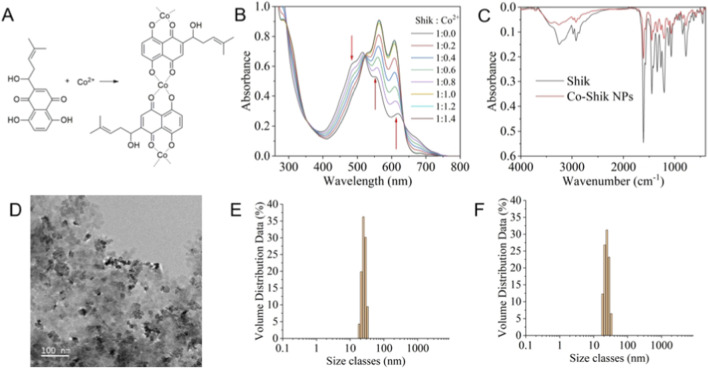
**(A)** Synthesis route of Co-Shik NPs; **(B)** UV absorption spectra of shik with different ratios of Co ions; **(C)** FT-IR spectra of shik and Co-Shik NPs; **(D)** Transmission electron microscopy (TEM) image of Co-Shik NPs; **(E)** Size distribution of Co-Shik NPs at pH 7.4 (PBS); **(F)** Size distribution of Co-Shik NPs in 100 mM NaCl solution.

A suspension of Co-Shik NPs was prepared via the reaction of cobalt chloride (CoCl_2_) with Shik (Figure 1). UV-Vis absorption spectroscopy revealed that when the molar ratio of Shik to Co ions reached 1:1, the absorption peak at 500 nm decreased, while the peaks at 550 nm and 615 nm significantly intensified, indicating the formation of a 1:1 coordination complex (Figure 1B). Fourier-transform infrared (FT-IR) spectroscopy analysis showed that upon coordination with Co, two characteristic peaks of Shik at 628.91 and 907.74 cm^−1^ completely disappeared (Figure 1C). The O-H stretching vibration peak exhibited a significant redshift from 3,251 to 3,258 cm^−1^, suggesting the involvement of hydroxyl oxygen atoms in coordination. Furthermore, the intensity of nearly all characteristic peaks (e.g., the C=O peak) was substantially reduced by approximately 60%–70%, demonstrating a systematic alteration in the electron cloud density of Shik due to the formation of coordination bonds with Co^2+^, resulting in a stable metal complex. Transmission electron microscopy (TEM) images showed that the nanoparticles possessed a uniform three-dimensional structure with an average particle size of 10 nm (Figure 1D). Under physiological condition at pH 7.4 and in 100 mM NaCl solution, Co-shik NPs exhibited a uniform particle size of approximately 20 nm with a PDI of 0.25 (<0.3), indicating excellent colloidal stability (Figures 1E,F). In contrast, after incubation in the wound microenvironment at pH 6.5, the PDI increased significantly to above 0.5, which was out of the valid distribution range, and no characteristic particle size peak could be detected. These results demonstrated that the nanoparticles underwent complete dissociation. The Co^2+^ release behavior of Co-shik NPs showed obvious pH responsiveness (Supplementary Figure S1). The cumulative Co^2+^ release rate of the pH 6.5 group (wound inflammatory microenvironment) was significantly higher than that of the pH 7.4 group (normal tissue environment), and the release rate was milder, which met the research requirements of promoting local wound healing and reducing systemic toxicity.

### 2.2 Cellular safety of Co-Shik NPs

**FIGURE 2:**
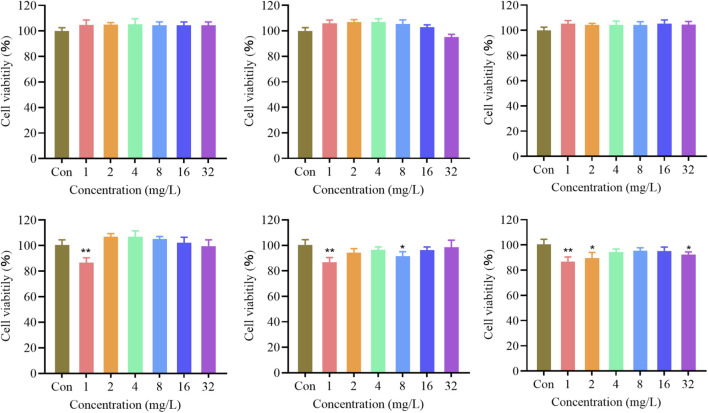
Viability of L929 cells treated with Co-Shik NPs at various concentrations; Viability of L929 cells treated with CoCl_2_ at various concentrations; Viability of L929 cells treated with Shik at various concentrations; Viability of L929 cells treated with 250 μM H_2_O_2_ and Co-Shik NPs at various concentrations; Viability of L929 cells treated with 250 μM H_2_O_2_ and CoCl_2_ at various concentrations; Viability of L929 cells treated with 250 μM H_2_O_2_ and Shik at various concentrations. (n = 6, *p < 0.05, **p < 0.01).

We assessed the *in vitro* cytotoxicity of Co-Shik NPs using the CCK-8 assay. The results showed that Co-Shik NPs at a concentration of 16 mg/L exhibited no significant toxicity towards L929 cells, with a cell viability close to 100%, indicating good biocompatibility (Figure 2). None of the individual components showed obvious cytotoxicity at the same concentration (Figure 2). Co-Shik NPs and CoCl_2_ at 32 mg/L exhibited no cytotoxicity toward RAW264.7 cells and mouse epidermal keratinocytes. Shik at 32 mg/L showed cytotoxicity toward both RAW264.7 cells and mouse epidermal keratinocytes (Supplementary Figures S3 and S4).

To verify the intracellular antioxidant capacity of the Co-Shik NPs, L929 cells were stimulated with 250 μM H_2_O_2_. H_2_O_2_ treatment reduced cell viability to approximately 80%. However, upon the addition of Co-Shik NPs at concentrations ranging from 16 mg/L down to 1 mg/L, cell viability was restored to 100% (Figure 2). This demonstrates that Co-Shik NPs can effectively scavenge intracellular reactive oxygen species, protecting cells from oxidative stress damage and exhibiting significant antioxidant activity.

### 2.3 Scavenging of intracellular reactive oxygen species by Co-Shik NPs

**FIGURE 3:**
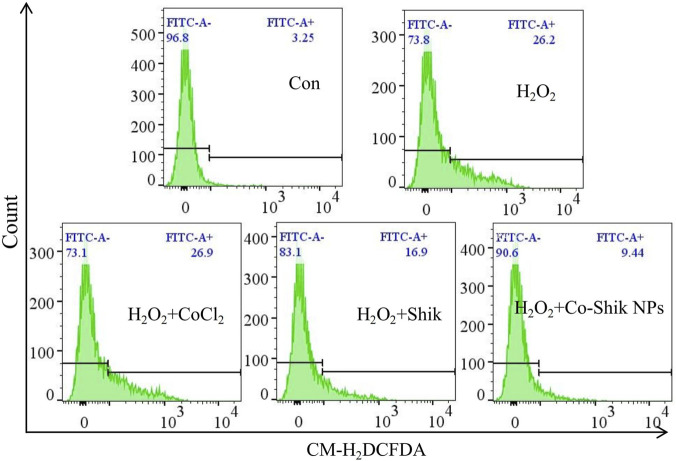
The effects of CoCl_2_, Shik, and Co-Shik NPs on ROS production in L929 cells stimulated with 250 μM H_2_O_2_ (n = 3).

Hyperglycaemia in diabetic patients leads to a significant increase in reactive oxygen species (ROS) levels, triggering mitochondrial ROS production and subsequent impairment of mitochondrial function. We employed the CM-H_2_DCFDA probe, which labels ROS with green fluorescence, to evaluate the intracellular antioxidant performance of the Co-Shik NPs (Figure 3). The results showed that H_2_O_2_ treatment increased the intracellular ROS level in L929 cells to 26.2%. Treatment with CoCl_2_ did not significantly alter the ROS level compared to the H_2_O_2_ group. In contrast, treatment with Shik and Co-Shik NPs reduced ROS levels to 16.9% and 9.44%, respectively. In Co-Shik NPs, cobalt ions coordinate with phenolic hydroxyl groups of shikonin. This coordination changes the redox state of cobalt and produces a synergistic antioxidant effect. The nanoscale structure also enhances aqueous dispersion and stability, leading to efficient ROS scavenging activity. Free cobalt chloride ions do not have these effects.

### 2.4 Inhibition of ROS-induced apoptosis by Co-Shik NPs

**FIGURE 4:**
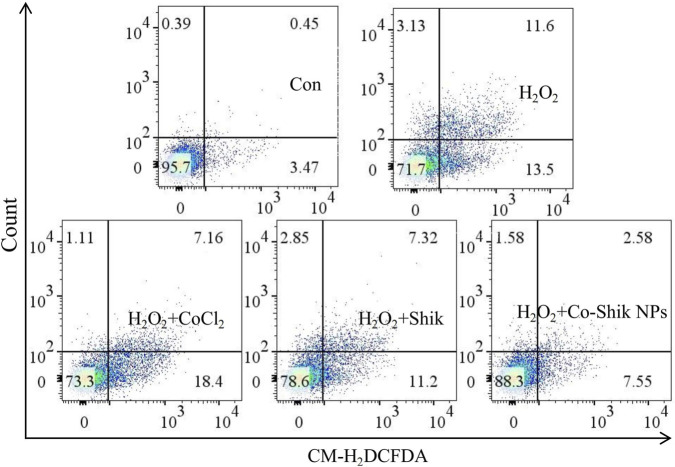
The effects of CoCl_2_, Shik, and Co-Shik NPs on apoptosis in L929 cells stimulated with 250 μM H_2_O_2_ (n = 3).

Stimulation with H_2_O_2_ induces excessive intracellular ROS generation, leading to oxidative stress damage and ultimately triggering apoptosis. To evaluate the inhibitory effect of Co-Shik NPs on ROS-mediated apoptosis, this study utilised Annexin V-FITC/Propidium Iodide (PI) double staining for flow cytometric analysis. The experimental results showed that the apoptosis rate of L929 cells in the H_2_O_2_ treatment group significantly increased to 25.1%, indicating successful induction of the apoptotic pathway by oxidative stress (Figure 4). Notably, the apoptosis rate in the CoCl_2_ treatment group (24.8%) showed no significant difference compared to the H_2_O_2_ group, suggesting that cobalt ions alone have no obvious inhibitory effect on ROS-induced apoptosis. In stark contrast, the Shik treatment group reduced the apoptosis rate to 18.52%, while the Co-Shik NPs group further significantly decreased it to 10.31%. This gradient protective effect indicates that Shik itself possesses certain anti-apoptotic capabilities, and the Shik-cobalt complex constructed via nanotechnology exhibits stronger biological efficacy.

### 2.5 Promotion of cell migration by Co-Shik NPs

**FIGURE 5:**
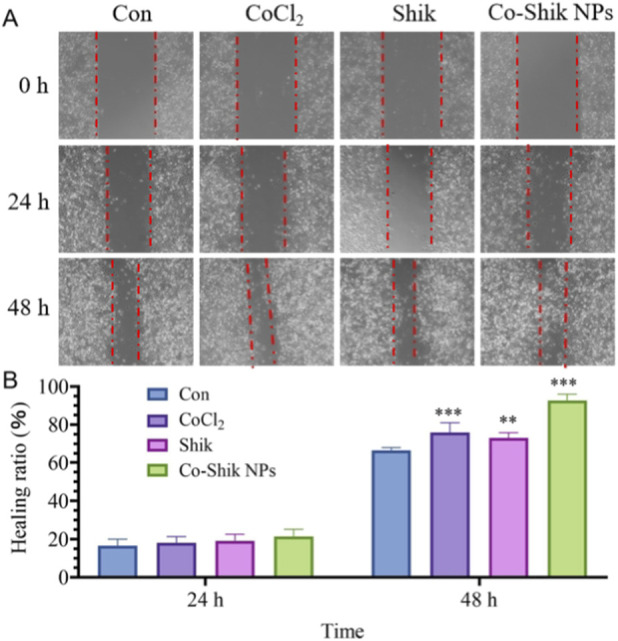
**(A)** Scratch wound images of L929 cells after 24 and 48 h of incubation with CoCl_2_, Shik, and Co-Shik NPs (n = 3); **(B)** Quantitative analysis of scratch wounds in L929 cells treated with CoCl_2_, Shik, and Co-Shik NPs for 24 and 48 h (n = 3, compared with control, **p < 0.01, ***p < 0.001).

We assessed the effects of different concentrations of CoCl_2_, Shik, and Co-Shik NPs on the migration ability of L929 cells using a scratch wound assay. Images were captured at fixed locations at 24 and 48 h post-scratch, and the cell migration area and wound closure rate were calculated using ImageJ software (Figure 5). The results showed that at the 24-h time point, the wound closure rate in the Co-Shik NPs group was slightly higher than that of the control group, but the difference was not statistically significant (p > 0.05). By 48 h, the group treated with 1 mg/L Co-Shik NPs exhibited a significant (p < 0.01) pro-migratory effect, achieving a wound closure rate of approximately 95%. In comparison, the closure rates for groups treated with CoCl_2_ or pure Shik at the same concentration were both below 80%. Co-Shik NPs can effectively promote the migration of L929 cells and accelerate gap closure. Their wound-healing promoting effect is significantly superior to that of the individual components, CoCl_2_ or Shik, suggesting a synergistic effect arising from the nano-scale composite formation of Shik and cobalt ions.

### 2.6 Promotion of diabetic wound healing in mice by Co-Shik NPs

**FIGURE 6:**
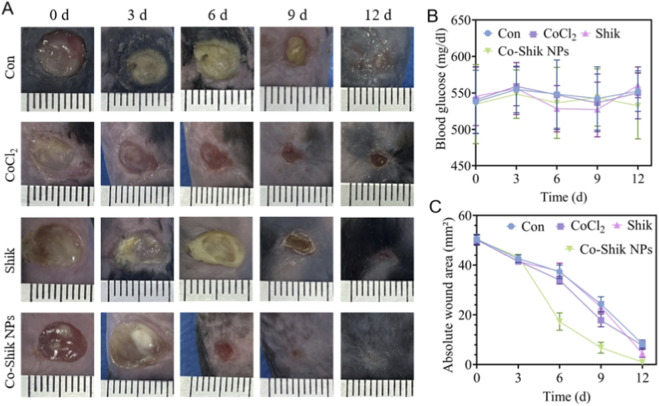
**(A)** Photographs of wound healing in different treatment groups on days 0, 3, 6, 9, and 12; **(B)** Blood glucose levels of mice during the experiment; **(C)** Wound healing rates in different treatment groups. (n = 6).

This study utilised a streptozotocin (STZ)-induced diabetic C57BL/6 mouse model to systematically evaluate the promoting effect of Co-Shik NPs on chronic wound healing. By continuously monitoring blood glucose levels (Figure 6B), we ensured that all experimental animals maintained a stable hyperglycaemic state (blood glucose >300 mg/dL) throughout the study period, providing a reliable animal model foundation for diabetic wound healing research. Dynamic monitoring of the wound healing process (Figure 6A) revealed that the Co-Shik NPs treatment group exhibited significant healing advantages at all time points. On day 3 of intervention, wound areas decreased in all groups, but inter-group differences were not yet statistically significant. By day 6, the wound healing rate in the Shikonin-Co nanoparticle group reached 65.37%, significantly higher than that in the CoCl_2_ group (32.15%) and the shikonin group (41.28%) (Figure 6C). This healing advantage further expanded with prolonged treatment. The healing rate in the nanoparticle group was 86.74% by day 9, and its wound contraction rate was 16.84% higher than other groups by day 12. The excellent wound-healing performance of Co-Shik NPs may stem from their nanostructure improving the bioavailability of the active components, allowing more effective penetration into the wound site. The synergistic action of Shik and cobalt ions enhances antioxidant capacity, effectively clearing excess reactive oxygen species (ROS) in the diabetic wound microenvironment and mitigating oxidative stress damage.

### 2.7 Reduction of early inflammatory response in diabetic mouse wounds by Co-Shik NPs

**FIGURE 7:**
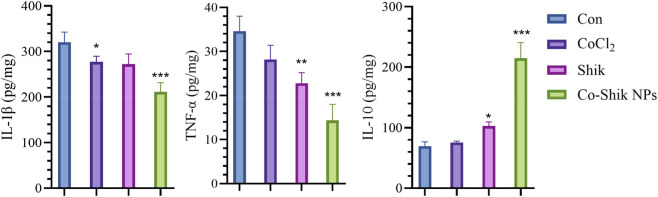
Concentrations of IL-1β, TNF-α, and IL-10 in wound tissues from different treatment groups measured by ELISA. (n = 3, *p < 0.05, **p < 0.01, ***p < 0.001).

To assess the anti-inflammatory mechanism of Co-Shik NPs in the diabetic wound healing process, we measured inflammatory cytokine levels in mouse skin tissue on post-operative day 3. The results showed that the Co-Shik NPs treatment group exhibited significant advantages in inflammatory regulation. Regarding pro-inflammatory factors, the IL-1β content in the nanoparticle group was 212.34 pg/mg, significantly (*p < 0.001) lower than the 310.29 pg/mg in the control group (Figure 7). TNF-α levels showed a similar trend, being markedly (*p < 0.001) lower in the nanoparticle group (12.35 pg/mg) compared to the control group (32.05 pg/mg) (Figure 7). Notably, the expression level of the anti-inflammatory factor IL-10 in the Co-Shik NPs group reached 215.62 pg/mg, significantly (*p < 0.001) higher than that in the control group. A hallmark of the diabetic wound microenvironment is a persistent inflammatory state, characterised by excessive expression of pro-inflammatory factors and relative deficiency of anti-inflammatory factors. Co-Shik NPs effectively break this inflammatory imbalance through bidirectional regulation of cytokine expression. This precise inflammatory modulation ability, combined with its excellent antioxidant properties, constitutes the core mechanism by which Co-Shik NPs promote diabetic wound healing.

### 2.8 Promotion of tissue regeneration, angiogenesis, and collagen production in diabetic mouse wounds by Co-Shik NPs

**FIGURE 8:**
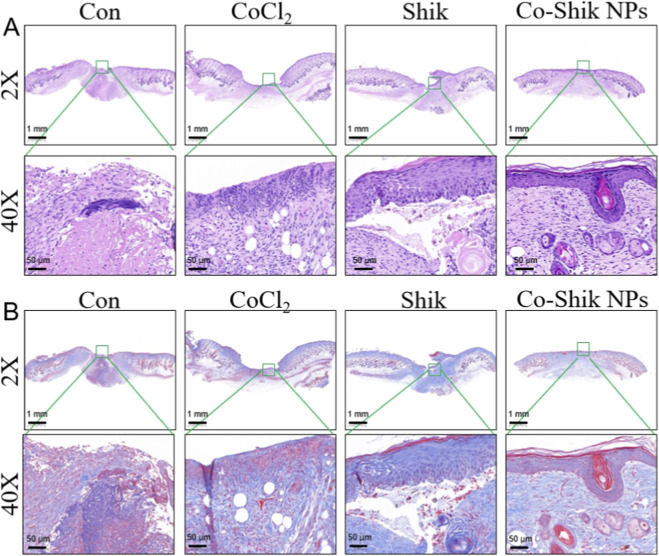
**(A)** H&E stained images of wound tissues from the CoCl_2_, Shik, and Co-Shik NPs groups; **(B)** Masson’s trichrome stained images of wound tissues from the CoCl_2_, Shik, and Co-Shik NPs groups. (n = 3).

Re-epithelialisation and granulation tissue formation are critical for wound healing. Histomorphological analysis revealed that on day 12, the Co-Shik NPs group displayed a more intact epidermis, denser granulation tissue, and even nascent hair follicles, in contrast to the discontinuous epidermis and loose granulation tissue in the Con group. The average epidermal thickness was also significantly higher in the Co-Shik NPs group (Figure 8A). Masson’s trichrome staining (Figure 8B) further demonstrated that Co-Shik NPs enhanced collagen deposition and induced denser, more regularly arranged collagen fibres, thereby improving extracellular matrix reconstruction and mechanical strength.

As shown in Supplementary Table S1 and Supplementary Table S2, semi-quantitative histological scoring further supported these findings. The Con group maintained a relatively normal structure, while CoCl_2_ induced obvious tissue damage. Shik treatment only partially alleviated such injury. In contrast, Co-Shik NPs significantly restored epithelial integrity, reduced inflammation and edema, and regularized collagen arrangement with the lowest scores. Collectively, these results demonstrate that Co-Shik NPs effectively promote wound healing by enhancing re-epithelialisation, granulation tissue maturation, and ordered collagen deposition, which may be attributed to improved inflammation, oxidative stress resistance, and angiogenesis.

**FIGURE 9:**
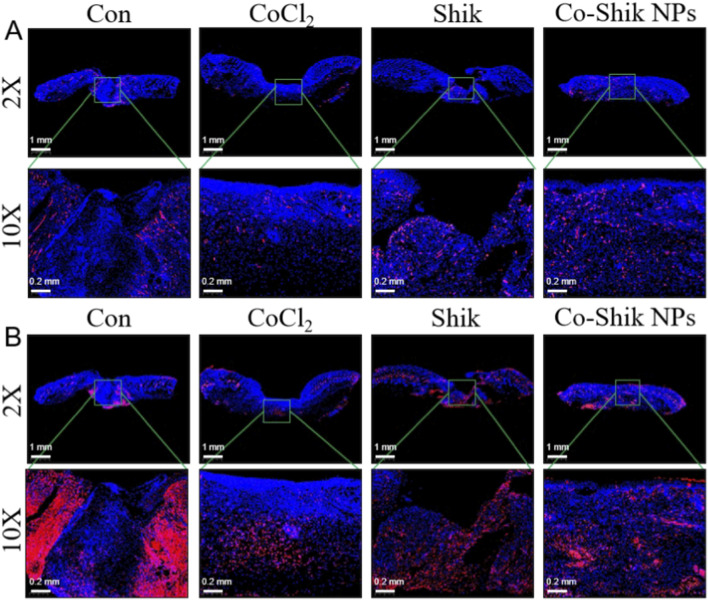
**(A)** CD31 immunohistochemistry of wound tissues from the CoCl_2_, Shik, and Co-Shik NPs groups; **(B)** VEGF immunohistochemistry of wound tissues from the CoCl_2_, Shik, and Co-Shik NPs groups. (n = 3).

CD31 immunohistochemical results (Figure 9A; Supplementary Figure S6) showed that the expression level of CD31 at the wound site was higher in both the CoCl_2_-treated group (108.67 ± 14.19) and the Shik-treated group (308.67 ± 61.41) than in the control group (100.00 ± 11.55). Notably, the expression level of CD31 in the Shik-Co NPs composite treatment group (360.00 ± 72.25) was significantly higher than that in the CoCl_2_ alone and Shik alone groups. VEGF immunohistochemical results (Figure 9B; Supplementary Figure S6) showed a similar trend. The expression level of VEGF at the wound site was higher in both the CoCl_2_-treated group (140.33 ± 8.21) and the Shik-treated group (249.67 ± 26.24) than in the control group (100.00 ± 17.32), and the VEGF expression level in the Shik-Co NPs group (278.67 ± 26.60) was also higher than that in the CoCl_2_ alone and Shik alone groups. These results indicated that both CoCl_2_ and Shik could promote the expression of CD31 and VEGF at the wound site to a certain extent, while combined treatment with Shik-Co NPs could further upregulate the expression levels of CD31 and VEGF, suggesting that the composite system exerts a synergistic effect in promoting angiogenesis and accelerating wound repair.

## 3 Conclusion

This study successfully constructed Shik-cobalt coordinated nanoparticles and systematically evaluated their therapeutic potential in diabetic wound healing. Fourier-transform infrared spectroscopy and ultraviolet-visible absorption spectroscopy confirmed the formation of stable nanoparticles with a 1:1 ratio of Shik to cobalt ions. *In vitro* experiments demonstrated that these nanoparticles showed no significant toxicity to L929 cells at a concentration of 16 mg/L, effectively scavenged intracellular reactive oxygen species, and significantly inhibited ROS-induced apoptosis. Co-Shik NPs effectively modulated the inflammatory state of the wound microenvironment by downregulating the pro-inflammatory factors IL-1β and TNF-α while upregulating the anti-inflammatory factor IL-10. Histological analysis further confirmed that the nanoparticle group promoted more complete epidermal structure formation and denser collagen fibre deposition, enhancing re-epithelialisation and granulation tissue maturation. Through synergistic mechanisms involving antioxidant, anti-inflammatory, and pro-regenerative effects, Co-Shik NPs significantly promoted diabetic wound healing. The conceptual advance of this work lies in the construction of Co-Shik self-assembled nanoparticles that integrate the biological functions of shikonin and Co^2+^. This nanosystem promotes type 1 diabetic wound healing primarily via enhanced antioxidant effects, providing a promising strategy for diabetic wound management.

## 4 Materials and methods

### 4.1 Experimental materials and reagents

CoCl_2_ and Shik were purchased from Shanghai Titan Scientific Co., Ltd. L929 cells and corresponding culture media were obtained from Shangcheng Beina Chuanglian Biotechnology Co., Ltd. All water used was double-distilled water.

### 4.2 Experimental animals

Specific pathogen-free (SPF) male C57BL/6 mice, aged 8–10 weeks and weighing 18–22 g, were used for the animal models. They were purchased from Sikebeisi Biotechnology Co., Ltd. All animal experiments were approved by the Biomedical Ethics Committee of Pingdingshan University and conducted in accordance with the university’s ethical standards for laboratory animals.

### 4.3 Preparation of Co-Shik NPs

1 mmol of Shik was dissolved in 10 mL of ethanol and mixed with an aqueous solution containing 1 mmol of CoCl_2_. The mixture was stirred at room temperature for 6 h. After the reaction, the mixture was centrifuged at 10,000 rpm for 5 min. The precipitate was collected and washed three times with deionized water to obtain the target product.

### 4.4 Characterisation of Co-Shik NPs

Ultraviolet-visible (UV-Vis) absorption spectra were recorded using a UV-Vis spectrophotometer (PE lambda 750) to analyse the optical absorption properties. Chemical structure characterisation was performed using a Fourier-transform infrared (FT-IR) spectrometer (Bruker Vertex 70). Detection of characteristic IR absorption peaks verified the structure of Shik and potential chemical interactions. Morphology was observed using a scanning electron microscope (ZEISS Gemini SEM 300), which provided direct visualisation of the nanoparticle surface morphology, particle size distribution, and aggregation state.

### 4.5 Cytotoxicity test

Cells were seeded in a 96-well plate at a density of 1 × 10^4^ cells per well (with 100 μL of culture medium per well). After 24 h of incubation, the original medium was removed and replaced with 200 μL of solutions containing different concentrations of CoCl_2_, Shik, or their nanoparticles (n = 4). Following another 24-h incubation, 100 μL of 10% CCK-8 solution was added to each well, and the plate was incubated for 1 h. Complete culture medium and pure water were used as negative and blank controls, respectively. Absorbance was measured at 450 nm using a microplate reader.

### 4.6 Cell scratch/wound healing assay

L929 cells were seeded in 6-well plates at a density of 6 × 10^5^ cells per well and cultured for 48 h until a confluent monolayer formed. A scratch was made in the monolayer using a 100 μL pipette tip, followed by two washes with PBS. The medium was then replaced with 2 mL of solutions containing different concentrations of CoCl_2_, Shik, or Co-Shik NPs (n = 3). Cell migration was observed and photographed at specific time points using an inverted fluorescence microscope.

### 4.7 Intracellular ROS scavenging test

Cells were seeded at an appropriate density in 12-well plates and cultured for 24 h. Subsequently, they were treated with 1 mg/L solutions of CoCl_2_, Shik, or Co-Shik NPs, co-incubated with 250 μM H_2_O_2_ for 24 h. The cells were then collected, washed twice with PBS, and incubated with 10 μM CM-H_2_DCFDA probe in the dark for 30 min. After two additional PBS washes, the cells were resuspended and analysed using flow cytometry to measure the fluorescence intensity, reflecting intracellular ROS levels. Data were processed using FlowJo software.

### 4.8 Cell apoptosis test

Cells were seeded at an appropriate density in 6-well plates and cultured for 24 h. They were then treated with 1 mg/L solutions of CoCl_2_, Shik, or Co-Shik NPs, co-incubated with 250 μM H_2_O_2_ for 24 h. After collection and two PBS washes, the cells were resuspended in 195 μL of Annexin V-FITC binding buffer. Then, 5 μL of Annexin V-FITC and propidium iodide (PI) were added, followed by incubation in the dark for 30 min. The cells were washed twice with PBS, resuspended, and analysed by flow cytometry. The results of Annexin V-FITC and PI double staining were analysed using FlowJo software to distinguish early apoptotic, late apoptotic, and necrotic cells.

### 4.9 Establishment of type I diabetic mouse model

After 1 week of acclimatisation, C57BL/6 mice were fasted for 4 h and weighed. Type I diabetes was induced by intraperitoneal injection of streptozotocin (50 mg/kg/day) for 5 consecutive days. During and 48 h after the injections, mice were given 10% sucrose water to prevent hypoglycaemia. Blood glucose levels were monitored from the tail vein starting 1 week after the first injection. Mice with blood glucose levels persistently ≥300 mg/dL for 2 weeks were considered successful models. After successful anaesthesia, the hair on the mouse back was removed under sterile conditions. Two symmetrical full-thickness skin wound areas were created on the back using an 8 mm biopsy punch. CoCl_2_, Shik, or Co-Shik NPs was topically applied to the wounds. The dosage for animal experiments was set according to relevant report (Hu et al., 2022). Co-Shik NPs solution was prepared at a concentration of 1 mg/L, and 250 μL was topically applied to the wound area each time, resulting in a dose of approximately 250 μg per administration. Once the fluidity decreased, the wounds were covered and secured with 3 M semi-permeable film dressings, which were changed every other day. Wound photographs were taken on days 0, 3, 6, 9, and 12 post-wounding. Wound area was measured using ImageJ software. The healing rate was calculated using the formula: Wound area (%) = (A_t_/A_0_) × 100%, where A_t_ and A_0_ represent the wound area on day t and day 0, respectively.

### 4.10 H&E and Masson’s trichrome staining

After treatment, mouse dorsal skin tissue was collected, rinsed with water, and fixed in 4% paraformaldehyde. Tissues were dehydrated through a graded series of ethanol (75%, 85%, 90%, absolute) and xylene, embedded in paraffin, and sectioned at 3 μm thickness. Sections were dried in an oven, dewaxed in xylene twice, and rehydrated through a descending ethanol series (absolute, 95%, 85%, 70%). For H&E staining, sections were stained with haematoxylin for 4–5 min, differentiated with acidified ethanol, and counterstained with eosin for 1 min. Sections were then dehydrated through an ascending ethanol series, cleared in xylene, and mounted for microscopic examination.

For Masson’s trichrome staining, following paraffin embedding and sectioning, sections were treated with ethylene glycol monoethyl ether acetate and rehydrated. They were stained with Weigert’s iron haematoxylin, differentiated with acidic ethanol differentiation solution, and rinsed. Subsequently, sections were blued in Masson’s bluing solution, stained with ponceau-acid fuchsin and aniline blue solutions, and rinsed. Finally, sections were dehydrated, cleared, mounted, and observed.

### 4.11 Inflammatory cytokine expression test

Skin tissue from day 3 was ground in liquid nitrogen, incubated with lysis buffer at 4 °C for 30 min, and centrifuged at 10,000 × g for 5 min. The supernatant was collected. Protein concentration was determined using the BCA method. Briefly, a BCA working solution was prepared. 20 μL of standards and samples were added to a 96-well plate, followed by 200 μL of the working solution. After incubation at 37 °C for 30 min, absorbance was measured at 562 nm to calculate protein concentration. Following the kit instructions, samples were analysed in triplicate. Absorbance was measured at 450 nm, and cytokine concentrations were calculated based on a standard curve, then normalised to pg per mg of protein.

### 4.12 Statistical analysis

Experimental data are presented as mean ± standard deviation (mean ± SD). Statistical analysis was performed using GraphPad Prism software (version 9.4.0). Comparisons between groups were conducted using one-way analysis of variance (ANOVA). The significance level was set at P < 0.05 (*P < 0.05, **P < 0.01, ***P < 0.001, ****P < 0.0001).

## Data Availability

The original contributions presented in the study are included in the article/Supplementary Material, further inquiries can be directed to the corresponding authors.
