# Supplementary material for: Shikonin and Co2+ self-assembled nanoparticles promote diabetic wound healing via antioxidant effects
Source: Front Chem. 2026 Apr 22;14:1785594. doi: 10.3389/fchem.2026.1785594 (PMC13144060; doi:10.3389/fchem.2026.1785594)
Supplement: Supplementary file 1 [file Supplementaryfile1.doc]

**Shikonin and Co^2+^ Self-Assembled Nanoparticles Promote Type 1 Diabetic Wound Healing via Antioxidant Effects**

**The Co^2+^ release behavior of Co-shik NPs in vitro.** The experimental reagents included Co-shik NPs synthesized in this study, PBS buffers at pH 7.4 and 6.5 (simulating normal tissue and wound inflammatory microenvironment, respectively), Co^2+^ standard stock solution, ultrapure water, and ultrapure nitric acid. The instruments selected were dialysis bags with a molecular weight cut-off (MWCO) of 3500 Da, a constant temperature water bath shaker, ICP-MS or AAS, etc. After pretreating the dialysis bags, Co-shik NPs were accurately weighed and dissolved in PBS buffers of the two pH values to prepare a suspension with a concentration of 200 μg/mL. A 5 mL aliquot of the suspension was injected into the dialysis bag, which was then sealed and immersed in 45 mL of PBS with the corresponding pH value, followed by shaking in a water bath at 37 ℃ and 100 rpm. At 0.5, 1, 2, 4, 8, 12, 24, and 48 h, 2 mL of the release medium was aspirated from each group and an equal volume of fresh PBS was added. After digestion with nitric acid, the Co²⁺ concentration in the samples was detected by ICP-MS or AAS. Each group had 3 parallel samples, and the experiment was independently repeated 3 times. The cumulative release rate was calculated according to the formula, and the kinetic curve was plotted.


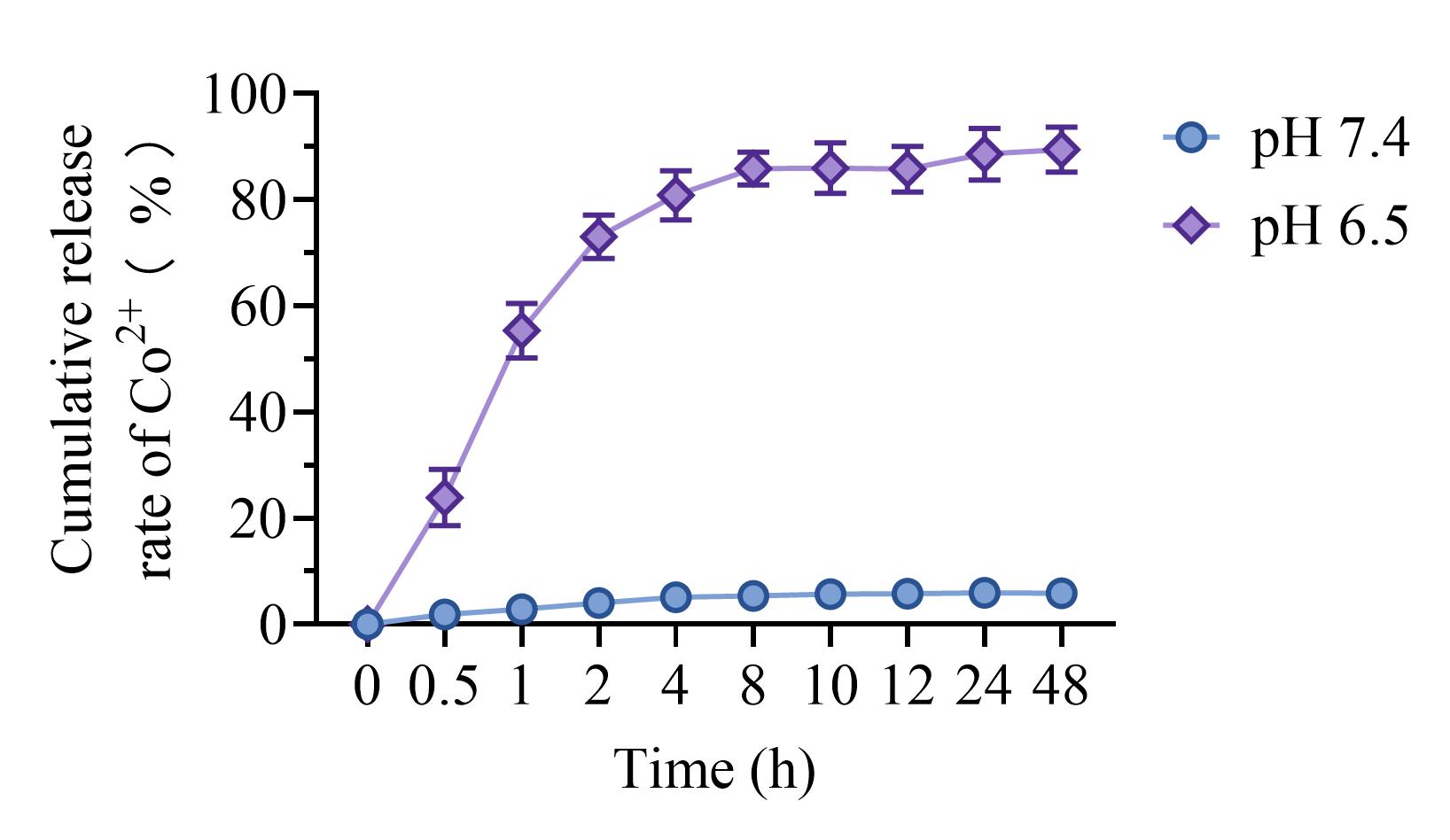


**Fig. S1.** The release rates of Co-shik NPs under different pH conditions (7.4 and 6.5).

**The maximum tolerated dose (MTD) of Co-Shik NPs.** SPF-grade C57BL/6 mice (6-8 weeks old, weighing 20±2g, half male and half female) were selected. After 3 days of adaptive feeding, the mice were randomly divided into 6 groups (6 mice per group), including a blank control group and Co-Shik NPs groups with gradient concentrations of 0.25-4.0 mg/mL. The reagents were prepared with sterile normal saline (freshly prepared and used immediately), and 250 μL was topically applied to the preset area on the back of each mouse once a day for 7 consecutive days, followed by 7 days of observation after drug withdrawal. The general condition, body weight, mortality and toxic symptoms of the mice were recorded. Based on the experimental design and preliminary experiment results, the maximum tolerated dose of Co-Shik NPs in this experiment will be 4.0 mg/mL.


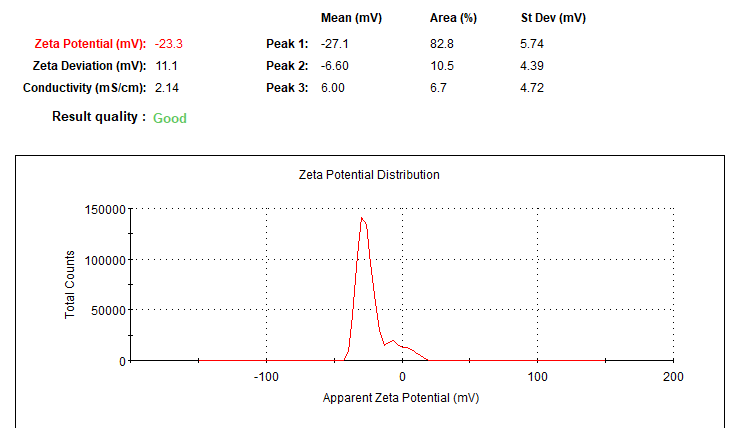


**Fig. S2.** Thezeta potential of Co-shik NPs.


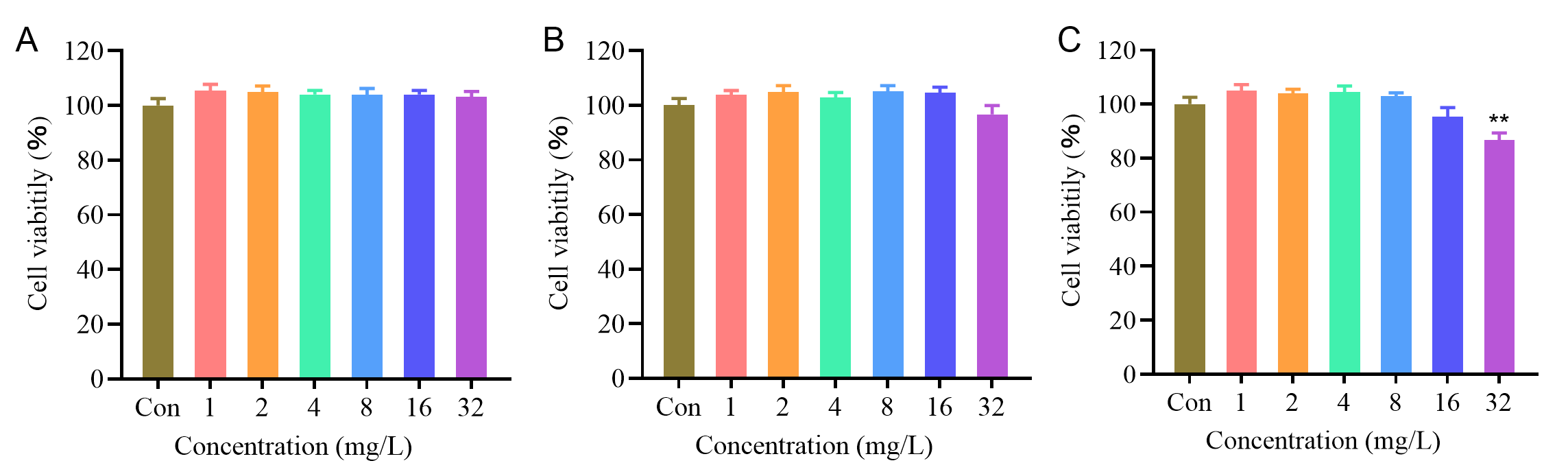


Fig. S3. (A) Viability of RAW246.7 cells treated with Co-Shik NPs at various concentrations; (B) Viability of RAW246.7 cells treated with CoCl₂ at various concentrations; (C) Viability of RAW246.7 cells treated with Shik at various concentrations. (n=6, **p<0.01)


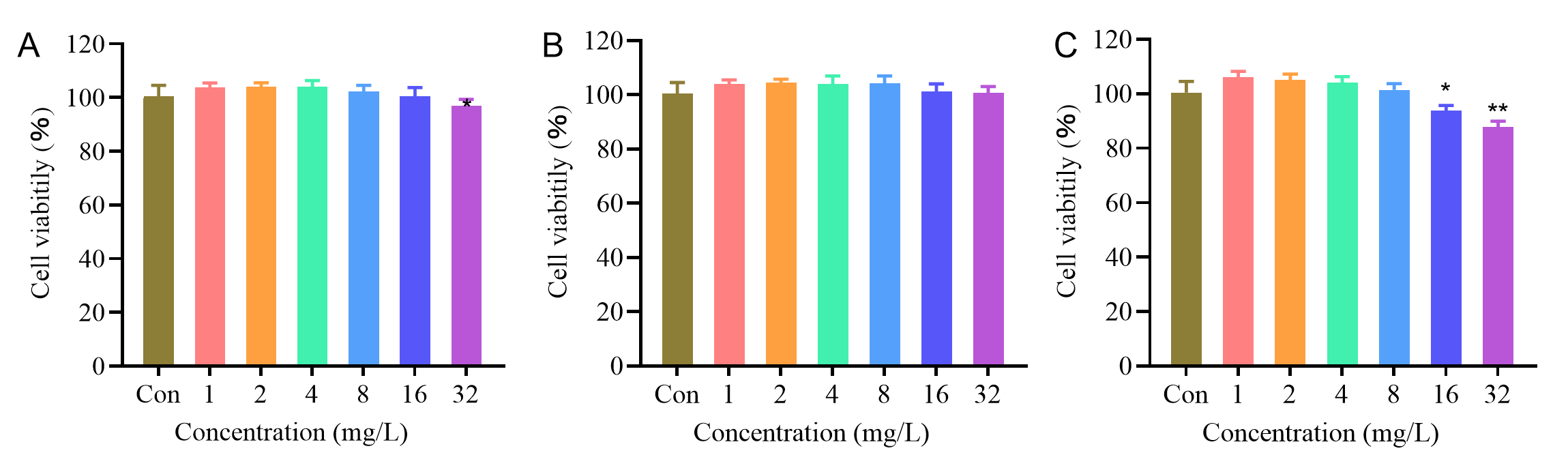


Fig. S4. (A) Viability of mouse epidermal keratinocytes cells treated with Co-Shik NPs at various concentrations; (B) Viability of mouse epidermal keratinocytes cells treated with CoCl₂ at various concentrations; (C) Viability of mouse epidermal keratinocytes cells treated with Shik at various concentrations. (n=6, *p<0.05, **p<0.01)


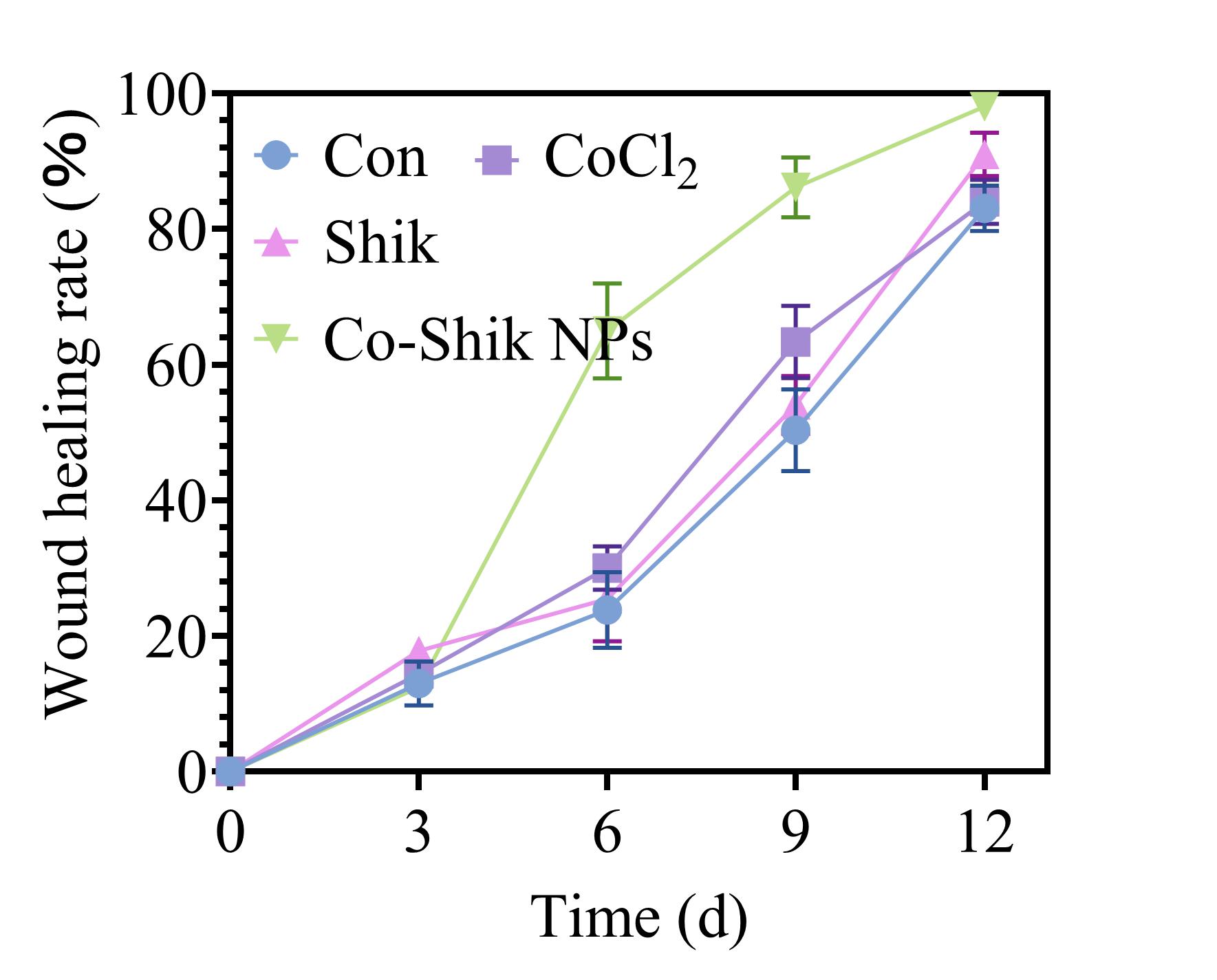


Fig. S5. Wound healing rates in different treatment groups (n=6).

Table S1. Standard for evaluation

| Score | Epithelial Integrity | Inflammatory Infiltration | Edema / Vacuolization | Collagen Arrangement |
| --- | --- | --- | --- | --- |
| 0 | Intact, normal structure | Absent / occasional | Absent | Dense and regular |
| 1 | Mild thinning, occasional interruption | Mild, <10/HPF | Mild scattered vacuoles | Mildly loose |
| 2 | Moderate thinning, multiple interruptions | Moderate, 10–20/HPF | Moderate vacuoles, focal edema | Moderately loose |
| 3 | Severe defect, extensive shedding | Severe, 20–30/HPF | Severe vacuoles, extensive edema | Severely loose |
| 4 | Complete loss, structural disarray | Extremely severe, >30/HPF | Extremely severe, massive liquefaction | Completely disorganized |

Table S2 Expected semi-quantitative results for each group

|  | Epithelial integrity | Inflammatory infiltration | Edema/vacuolation | Collagen arrangement |
| --- | --- | --- | --- | --- |
| Con | 4 | 4 | 4 | 4 |
| CoCl₂ | 3 | 3 | 4 | 3 |
| Shik | 1 | 2 | 2 | 1 |
| Co-Shik NPs | 0 | 1 | 0 | 0 |


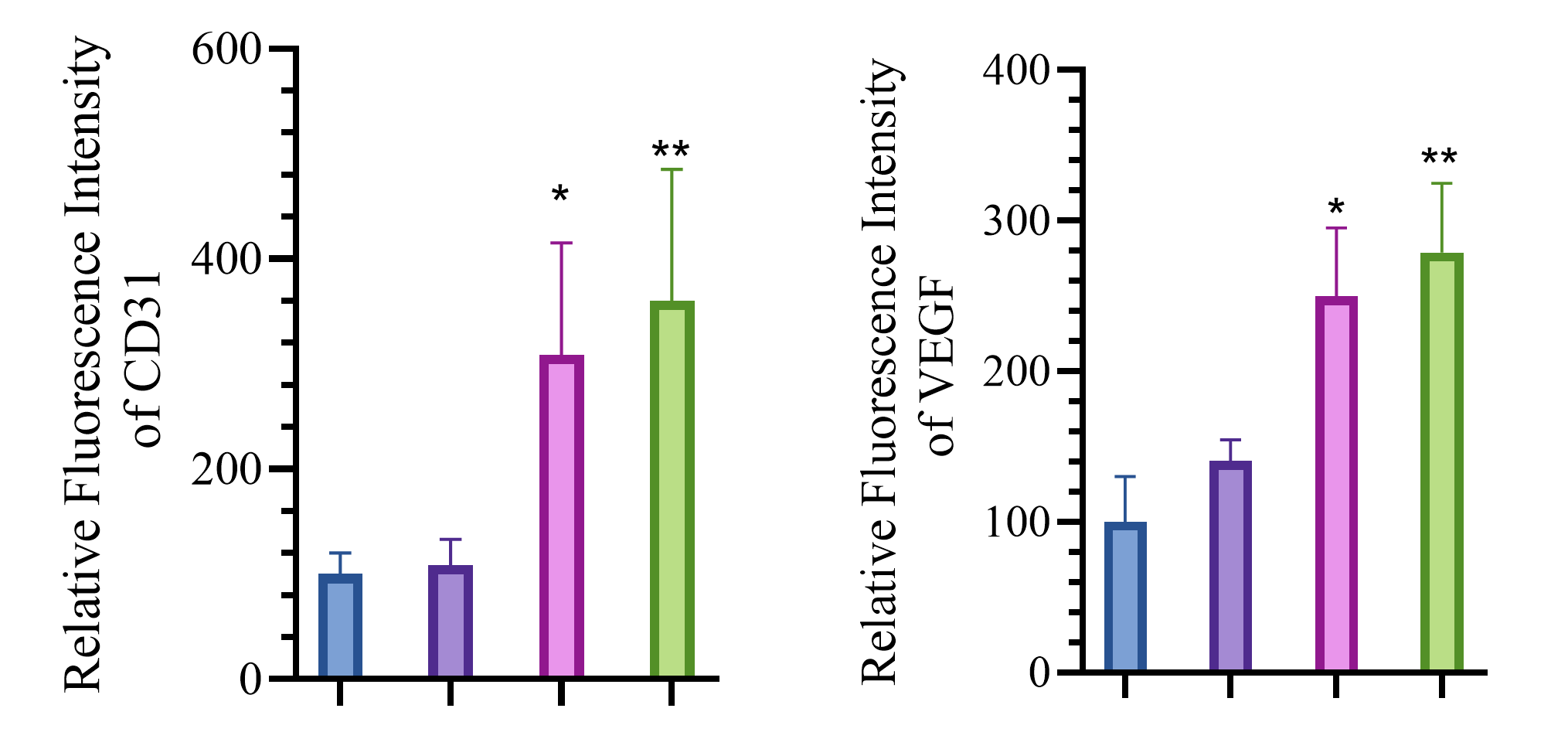


Fig. S6. Relative Fluorescence Intensity of CD31 and VEGF (n=3, *p<0.05, **p<0.01).
